# The assessment of new drugs for asthma and COPD: a Delphi study examining the perspectives of Italian payers and clinicians

**DOI:** 10.1186/s40248-016-0038-3

**Published:** 2016-01-27

**Authors:** Silvia Sommariva, Aureliano P. Finch, Claudio Jommi

**Affiliations:** 1 Centre for Research on Health and Social Care Management, Bocconi University, Milan, Italy; 2 Department of Pharmaceutical Sciences, Università del Piemonte Orientale A. Avogadro, Novara, Italy; 3 Università Bocconi - CERGAS, Via Roentgen, 1, 20136 Milano, Italy

**Keywords:** Asthma, COPD, Delphi

## Abstract

**Background:**

Asthma and chronic obstructive pulmonary disease (COPD) are disorders of the lungs characterized by airflow obstruction, inflammation and tissue remodeling. Management of patients with these diseases is complex and the improvement of diagnostic-therapeutic strategies represents a critical challenge for the healthcare system. In this context, investigating the criteria and information needed for an appropriate and effective evaluation of incoming treatment options is crucial to ensure that clinicians and policy-makers are provided with the best available evidence to make decisions aimed at improving patient outcomes. Therefore, the objective of this study was to investigate the degree of agreement among Health Technology Assessment (HTA) experts on issues crucial to the evaluation of new drugs for asthma and COPD and to appropriately manage the clinical pathway for patients.

**Method:**

This research was conducted using an e-Delphi technique organized in three subsequent rounds and involving a panel of ten experts (six regional and local payers and four clinicians). Panelists were asked to comment in written form on a set of statements, explaining qualitatively the extent to which they agreed or disagreed with the assertions. Statements were subsequently modified and resubmitted for assessment.

**Results:**

Panelists expressed their opinions during each round and, after round III, a consensus document was finalized. The degree of consensus was high among experts and concerned five main topics: (a) the need to address current unmet needs of patients with asthma or COPD, (b) the importance of further studies and real-life information in the evaluation of treatments, (c) existing evidence and evidence needed to assess drugs, (d) critical issues in obtaining a positive evaluation from regional and local authorities for new treatments to be included in regional formularies and to have an important place in therapeutic categories, and (e) the major obstacles to the appropriate administration of drugs and management of patients.

**Conclusion:**

The final document highlights that no proof of difference among drugs exists, that evidence on final endpoints (and particularly on mortality) should be strengthened and that actions regarding risk factors, appropriate diagnosis, patient staging and adherence to therapy are particularly important for a better clinical management.

**Electronic supplementary material:**

The online version of this article (doi:10.1186/s40248-016-0038-3) contains supplementary material, which is available to authorized users.

## Background

Asthma and chronic obstructive pulmonary disease (COPD) are disorders of the lungs characterized by airflow obstruction, inflammation and tissue remodeling. Although these diseases represent a major health problem, they are still largely underdiagnosed and undertreated [1].

Recent estimates suggest an asthma prevalence of 6.9 % in the Italian population, with an increasing pattern in recent years [2]. Approximately 3 % of the Italian population had COPD in 2011 [3], which according to the technical-scientific Agency of the Italian National Health Services (Agenas) is likely to underestimate the magnitude of the phenomenon [4]. According to the World Health Organization (WHO), the total number of COPD-related deaths is projected to increase by more than 30 % over the next 10 years [5].

In addition to the increasing epidemiological burden of the disease, the management of affected patients is complex; improving diagnostic-therapeutic pathways represents a challenge for the healthcare system [6–8]. Identifying the most critical points in the clinical pathway and the extent to which the system can be improved are, therefore, fundamental issues on which experts must reach consensus. Moreover, investigating the criteria and information needed for an appropriate and effective evaluation of incoming treatment options is crucial to ensure that clinicians and policy-makers are provided with the best available evidence in order to make decisions aimed at improving patient outcomes.

The primary objective of this study was to investigate the degree of agreement and collect the opinions of a panel of experts about a selection of issues crucial to the evaluation of new treatments for patients with asthma and COPD. Specifically, the statements focused on critical aspects of the diagnostic phase of these diseases, correct disease staging, role of real life evidence, boosting patient compliance to treatments and transferability of study results in national contexts.

## Method

The study utilized a Delphi methodology, a technique used to facilitate information-gathering and communication on complex issues [9]. As presented in the literature, the process was characterized by three aspects: anonymity of study participants during the process, in order to avoid influences within the group; iteration, to allow participants to change their opinion from one round to another; and controlled feedback, meaning that panelists were provided with the results of previous rounds [10–13].

For the purposes of this study, an e-Delphi technique (e-mail based; [14, 15]) consisting of three rounds and involving a panel of ten experts was employed. Experts were selected on the basis of their expertise in the processes of HTA evaluation of new treatments for asthma and COPD. Their professional roles varied from institutional and managerial to clinical: three experts represented regional-level healthcare authorities, three panelists worked for local health authorities, and four were clinical experts, some with roles in national scientific societies. We excluded representatives of the National Drugs Agency (Agenzia Italiana del Farmaco-AIFA) from the panel of experts, because (i) AIFA negotiates price and reimbursement and is less involved in governing and managing clinical pathways, (ii) new drugs’ price and reimbursement criteria and information sources are set by law [16], and (iii) regional governments and local authorities are the actual payers.

Panelists were asked to comment on a set of eight statements presented in a .docx document (Italian language), explaining in a qualitative way the extent to which they agreed or disagreed with the assertions. Statements were subsequently modified to incorporate comments, and resubmitted for assessment to each of the panelists independently. As suggested by Keeney et al. [14], similar statements were reduced to a single encompassing statement, maintaining the original wording to the extent possible. In case of contrasting opinions, both views were reported in the statement specifying that panelists differed in their views on that specific assertion. The statements reported in this document are, therefore, not the original ones submitted for panelists’ comment but the final statement on which experts intervened and finally reached consensus on.

The original statements were constructed following a non-systematic review of the literature regarding two issues that are particularly critical for the stakeholders: evaluation of drugs by Italian HTA agencies and implementation of diagnostic-therapeutic pathways in the Italian National Health Service (NHS). The role of clinical pathways in the field of asthma and COPD has been widely recognized as crucial for improving patient outcomes [17, 18] and this is particularly relevant in the Italian context, where the importance of diagnostic-therapeutic paths as a tool to ensure application of clinical guidelines [19, 20]) has received extensive attention. To this end, the analysis of issues around the definition of care pathways we conducted in this study represents an important step toward compliance with international guidelines. Moreover, analyzing and promoting discussion over criteria and evidence deemed necessary by payers to evaluate new treatments is crucial to balance increasingly scarce resources with the sustainability of clinical pathways [21]. Therefore, statements were also included to address this issue from the perspective of Italian payers.

## Results

The process toward consensus was fluid, as no major disagreement was encountered since the first round. Comments by panelists mostly added complementary information to the statements provided or helped in clarifying the statements. For example, one panelist stressed the importance of primary prevention, which was not included in the first document draft; all responders then agreed to include actions aimed at reducing risk factors as a priority. Consensus was reached on eight statements encompassing five main topics: unmet needs of patients with asthma or COPD, criteria for the evaluation of new treatments, existing evidence and evidence needed to assess drugs, critical issues in obtaining a positive evaluation from regional and local authorities for new treatments to be included in the regional formularies, and obstacles for the appropriate administration of drugs. Prevention, diagnosis and staging of patients were identified as critical points of the clinical pathway, which should be addressed by further actions aimed at educating patients and clinicians, collection and analysis of relevant population data, and at promoting adherence to guidelines. Existing and desirable evidence regarding therapeutic treatments was discussed, emphasizing the importance of real-life information for these complex diseases. Adherence to therapy was also highlighted as a crucial area in which improvement is needed.

The document presented to panelists had a ‘Background’ section, introducing key data on the two diseases and some of the relevant drugs currently reimbursed in Italy (see list in Additional file 1). A second section included the eight statements and some contextualization remarks. This section reported the English translation of the final consensus document on the statements reached after round III. Panelists expressed their opinions in each round and, after round III, a consensus document was finalized, incorporating changes to the original document.

### Delphi document – final version

According to the literature, asthma and COPD are often underdiagnosed [1, 22]. This is mainly due to three factors: i) the chronic and recurrent nature of these diseases requires a careful monitoring of patients to detect arising symptoms; ii) COPD is characterized by a discrepancy between symptoms and the degree of airflow obstruction; and iii) patients tend to get accustomed to symptoms, adapting their lifestyle to their new health status. Failure to recognize the symptoms of these diseases is therefore frequent among patients. For instance, COPD symptoms are sometimes mistakenly interpreted as asthma symptoms at the time of diagnosis [23–25]. As outlined by National Institute for Health and Care Excellence (NICE), it should be considered that the process of diagnosis for asthma differs in adults and children and also varies among adults and among children [26].

Inappropriate diagnosis and, consequently, inappropriate treatment, entails significant costs for the healthcare system. In particular, significant expenditures occur due to hospitalization [24, 27] and indirect costs in the form of “presenteeism” (presence at the workplace when sick) [28].

Given these findings we can state that:

**Table:** 

*Statement 1* *One of the most critical aspects of COPD and asthma is represented by the diagnosis and identification of target patients. However, considering that COPD and asthma are preventable diseases, it is important to invest resources not only to improve the diagnostic phase, but also on prevention and, particularly, on the recognition of risk factors. This requires the involvement of professionals, especially General Practitioners (GPs), in tailored training programs.* *With respect to the time of diagnosis, the most critical issues are: i) failure to distinguish between COPD and asthma; and ii) rare use of the spirometer (for reasons such as low utility attributed to the tool by patients and clinicians, complexity of result interpretation, waiting time, and out-of-pocket expenses).* *It is therefore necessary for healthcare organizations to invest in initiatives aimed at promoting prevention and early diagnosis, through a greater focus on risk factors, patient education, a multidisciplinary approach to the diseases and equity of access to services.* *Specifically there is need for: i) a shift from the prevalent model of watchful waiting to more proactive approaches to identify patients potentially at risk; ii) training for GPs and other health professionals, with specific reference to GOLD, GINA and BTS clinical guidelines among others [* 29 *–* 31 *] and the opinions of national scientific societies regarding risk factors (particularly tobacco smoke) and the use of the spirometer for a correct diagnosis and follow-up; iii) development and spread of the use of informatics tools to analyze the issue in terms of population data, in order to improve organizational planning and analysis of standards of assistance for different categories of patients; and iv) educational initiatives for patients, who often underestimate the importance of these diseases and do not pay attention to symptoms.*	

A correct staging of the diseases is needed to define the appropriate therapeutic scheme [29, 30]. Considering this, we can state that:

**Table:** 

*Statement 2* *During the diagnostic and therapeutic path of the patient with COPD or asthma, the most critical moment is the one of disease staging for the purposes of appropriate drug prescription or other therapeutic interventions. The staging process needs to include an effective management of co-morbidities and symptoms.* *Healthcare organizations should invest in actions aimed at the improvement of early staging of patients, in order to allow an efficient allocation of resources. Staging first requires a correct execution of pulmonary function tests and particularly of spirometry, conducted periodically and with the collaboration of different professional figures (pulmonologist, GP, nurse operating in primary care) to guarantee easier access to the test and correct interpretation of the results. Healthcare organizations can exercise organizational planning and control on this issue, requesting periodic reports from physicians without this translating into an excessive bureaucratic burden.*	

With respect to the available pharmaceutical options, a recent systematic review has demonstrated a substantial comparability of the efficacy of aclidinium bromide, tiotropium bromide and glycopyrronium bromide as maintenance therapy for COPD [32]. A second, less recent systematic review by Shepherd et al. [33] on the comparative efficacy of different inhaled corticosteroids in association with different long-acting beta-2 agonists, indicated a substantial similarity of the efficacy between these asthma treatments. The evidence, however, is not sufficiently robust and the most recent *real-life* data (PATHOS study; [34]) showed difference in the efficacy of fixed associations on intermediate endpoints. It can be consequently stated that:

**Table:** 

*Statement 3* *Given the evidence published to date, fixed associations of ICS/LABA available on the market for asthma and COPD present “no proof of difference”, at least with respect to the clinical outcomes found during registration trials, meaning surrogate endpoints (*e.g. *FEV1 at 24 h after administration). The evidence is however insufficiently robust to be able to confirm the presence of therapeutic equivalence between these drugs. More recent real-life studies (*e.g. *PATHOS study, [* 34 *]) show instead a difference between fixed associations when looking at intermediate endpoints (number of exacerbations and hospitalizations).*	

The literature has already compared survival outcomes between patients taking fixed combinations of corticosteroids administered by inhalation and LABA, and fixed combinations of corticosteroids administered by inhalation and short-acting muscarinic antagonist (SAMA) in patients with COPD. The data demonstrated superior survival outcomes for the former treatments over the latter [35]. A rather more recent study (trial TORCH; [36]) shows that a fixed salmeterol/fluticasone association does not result in a statistically significant reduction in mortality when compared to monotherapies or placebo. The evidence on mortality cannot be deemed sufficiently robust to draw conclusions on survival differentials. We can therefore conclude that:

**Table:** 

*Statement 4* *Given that pharmaceutical treatment for COPD is primarily aimed at the reduction of dyspnea to improve patients’ quality of life, and not to reduce mortality, there is no available evidence regarding the impact of different fixed associations on patients’ mortality. The TORCH* trial *[* 36 *] has missed the statistical significance on the primary outcome of mortality. The availability of such data would, however, be important for therapeutic decision-making.*	

Adherence to therapy is also fundamental for the optimal management of chronic respiratory diseases and to avoid the waste of resources. Several studies show the negative impact of low adherence to therapy on morbidity and mortality rates, number of hospitalizations, and spending, which is higher in the case of low adherence with therapies [36–38]. Consequently:

**Table:** 

*Statement 5a* *A most critical aspect of treatment for COPD and asthma is the adherence to therapeutic treatments. Data from the literature show a real-life adherence between 10 and 40 %,* versus *70 to 90 % in trials [* 39 *]. Higher adherence to therapeutic treatments can be achieved through educational and training activities, addressed to patients and caregivers, regarding the disease characteristics and device use [* 40 *–* 43 *]. Additional factors that can impair adherence are polytherapy, frequent administration, degree of perception of benefits and adverse events. Therefore, it would be appropriate to activate programs to monitor prescriptions and the use of therapy that allow intervention with patients showing poor adherence to treatments.* *Statement 5b* *Poor adherence is among the factors affecting the acutization of COPD exacerbations and poor management of asthma.*	

Given the strict inclusion/exclusion criteria of patients in clinical trials and in the study design, there are issues that are left unanswered by registration studies in the area of respiratory diseases. Some of these questions include: What is the real efficacy and safety of the treatments available in different patient subgroups? What is the effect of prolonged exposure to therapy? What is the real adherence rate? For this reason:

**Table:** 

*Statement 6* *For currently-available treatments as well as drugs in development, it is important to collect real-life evidence on their overall effects (clinical and economic) and, in particular, on the elements that affect adherence to therapy. Among these, a number of factors are of particular relevance: i) the number of drugs taken and frequency of administration; ii) the type of device used; and iii) the level of knowledge and education of the patient and the relationship s/he has with the physician.* *Real life evidence on adherence and the contribution of individual factors (specifically, the type of device used) can be gathered through observational studies, both prospective and retrospective, possible on a large scale, to be able to conduct analyses on homogeneous subgroups. Such studies can be conducted by GPs in cooperation with specialists, and using existing databases, on the condition that data cover a large portion of the Italian national territory and that the data are comprehensive and of good quality. Studies of this type conducted in the Italian context have for instance provided relevant insights on critical aspects effective management of asthma and COPD, pinpointing the problem of inhaler mishandling in real-life settings [* 44 ,45 *].* *Statement 7* *For a disease such as COPD, pragmatic trials are potentially useful to collect evidence as a proxy to real-life relevant endpoints. These studies allow for maximization of the generalizability of results and their applicability to clinical practice, which is particularly relevant for a complex disease like COPD, which often occurs with several co-morbidities.*	

Finally, given that evidence from certain studies could not be generalizable to other populations, due to pharmacogenomic differences, differences in settings and clinical therapeutic pathways in which new drugs are introduced, we could say that:

**Table:** 

*Statement 8* *Whenever problems of transferability of results from international studies are found, it is necessary to plan studies in the Italian context, in particular real-life studies. These analyses would allow for an evaluation of the epidemiological profile of patients affected, documentation of the diagnostic approach and evaluation of the treatment strategies and pathways followed.*	

## Discussion and conclusions

This study attempted to identify the most critical aspects of COPD and asthma treatment and discuss the extent to which current managerial and policy approaches can be improved. The results of the Delphi research highlight which information and actions are deemed relevant by clinicians and policy makers for an appropriate and effective evaluation of incoming treatment options. This has important implications for the stakeholders involved, who could benefit from these findings for the identification of critical information helpful for the effective evaluation of new treatments. The discussion of the critical steps in managing patients with these diseases allows clinicians, policy-makers and managers to identify priorities of intervention, and the industry to be able to offer supportive services addressing key concerns in the therapeutic decision process.

Panelists’ agreement on statements was very high from the very beginning of the Delphi Study. This may reflect a generalizable common view of payers (at regional and local levels) and clinicians on the two major topics investigated, i.e., information needs regarding drugs and clinical pathways. In addition, no significant differences were observed between subgroups of experts depending on their background.

There was, first, a general consensus that there is no proof of difference among the existing therapeutic alternatives, even if more recent real-world data show differential effects of drugs at intermediate endpoints (exacerbations and hospitalizations). Panelists also stressed that there is no evidence on mortality and that the availability of such evidence could support decisions on drug comparison and better resource allocation. Despite their complexity, pragmatic trials were considered very useful for collecting information that may be considered a proxy of real-world evidence.

From a managerial viewpoint, actions on risk factors, appropriate diagnosis, and patient staging were considered the most critical aspects. Local payers have emphasized the importance of a multidisciplinary approach to staging and treatment, through the cooperation of different healthcare professionals, while the importance of preventive medicine and early diagnosis through careful monitoring of higher risk patients was highlighted by clinicians. As a consequence, initiatives aimed at proactively identifying patients potentially at risk, training programs, especially for GPs (but within a multi-disciplinary approach), and educational initiatives for patients, were identified as the primary targets of public investments. Adherence to therapy was also considered a critical issue. Panelists highlighted that the frequency of drug administration, device use, polytherapy and drug safety profiles may strongly influence adherence. For this reason, they advocate for real-world evidence on adherence that should integrate data on drug risk-benefit profiles.

All these recommendations are useful both for payers, if they want to pursue efficiency in resource allocation, and for the industry, if it wants to appropriately integrate data available at market launch with post-marketing evidence and support payers’ initiatives to promote a better management of clinical pathways.

## Additional file

Additional file 1:
**Drugs for COPD/ Asthma treatment.** (DOCX 32 kb)
