# Supplementary material for: The assessment of new drugs for asthma and COPD: a Delphi study examining the perspectives of Italian payers and clinicians
Source: Multidiscip Respir Med. 2016 Jan 27;11:4. doi: 10.1186/s40248-016-0038-3 (PMC4730839; doi:10.1186/s40248-016-0038-3)
Supplement: Additional file 1: — Drugs for COPD/ Asthma treatment. (DOCX 32 kb) [file 40248_2016_38_MOESM1_ESM.docx]

**Additional file 1**

| ICS/LABA Drugs admitted for reimbursement in Italy for COPD treatment |
| --- |
| - salmeterol/fluticasone - budesonide /formoterol - indacaterol |
| ICS/LABA Drugs admitted for reimbursement in Italy for Asthma treatment |
| - salmeterol/fluticasone - budesonide /formoterol - formoterol/fluticasone - formoterol/beclometasone |
